# Operations on the Uterus and Appendages during Pregnancy

**Published:** 1912-03

**Authors:** W. C. Swayne

**Affiliations:** Professor of Obstetrics, University of Bristol; and Obstetric Physician, Bristol Royal Infirmary


### OPERATIONS ON THE UTERUS AND APPENDAGES DURING PREGNANCY.

BY

W. C. Swayne, M.D. Lond.,

*Professor of Obstetrics, University of Bristol; and Obstetric Physician, Bristol Royal Infirmary.*

It is commonly believed that the uterus is rendered by pregnancy so susceptible to either direct or indirect stimuli, that any kind of physical or mental shock will set up contractions and so lead to the evacuation of its contents. That this is not so in all cases, and that not only may surgical operations be safely undertaken without terminating pregnancy, but that operations on the uterus itself do not necessarily lead to the termination of pregnancy, is shown by the short series of cases which follows.

Pathological conditions may arise at any time during pregnancy in connection with the appendages, or the uterus itself, calling for immediate surgical intervention. Ovarian tumours form one class of these conditions, and may call for surgical intervention either in the form of immediate operation on account of accidents to them as the result of pregnancy, or of a more deliberate nature in order to avoid dangers liable to occur either later in pregnancy or during the puerperium.

Ovarian tumours may, for example, be so situated as to obstruct labour ; torsion of the pedicle in those not so situated may occur, and is as a matter of fact a common occurrence ; pain and pressure symptoms due to their rapid enlargement ; inflammation and suppuration ; or rupture of the cyst may occur in the course of pregnancy.

Labour may cause mechanical injury, torsion of the pedicle, or thrombosis of the vessels from stretching on the pedicle, and produce serious symptoms during the puerperium due to the same causes.

It has been shown (McKerron) that in all cases in which ovarian tumours accompany pregnancy the maternal mortality is high and that artificial methods of delivery, if they obstruct labour, *per vias naturales*, even if successful, are highly dangerous. The safest method in such cases is to remove the tumour, if it can be reached without too great difficulty.

In some cases this may be extremely difficult, and I have myself been compelled to perform Cæsarean section in one case before the tumour could be removed, owing to dense adhesions. Such a complication as twisted pedicle is liable to occur as the result of the displacement of the tumour by the enlarging uterus during pregnancy, or by its consequent sudden diminution during labour.

It is obvious that the earlier in pregnancy the presence of the tumour is discovered the easier will be the operation for its removal, since the presence of an enlarged uterus necessarily renders the operation more complicated.

In any case the recognition of the presence of an ovarian tumour in a pregnant woman should lead to its removal either immediately if acute symptoms are present, or with as little delay as is reasonably possible if the symptoms are not urgent, especially if the tumour be solid.

Myomata of the pregnant uterus, although they do not call so urgently for surgical intervention, should always be looked on with suspicion, since they also may produce serious complications. They invariably enlarge with a rapidity quite apart from the apparent enlargement due to increasing size of the uterus, and this enlargement may be so marked as to call for their removal on account of upward pressure on the thorax and interference with respiration. They may undergo degeneration, either "red" or myxomatous, or become œdematous.

If pedunculated, the pedicle may become twisted and the blood supply cut off, or if not pedunculated, but growing from one side of the fundus, they may cause acute axial torsion of the uterus; if situated in the lower segment of the uterus, they may obstruct labour, and necessitate Cæsarean section. Even if they do not give rise to any marked complication during pregnancy, they are liable to suffer considerable damage during the puerperal period, and during labour may lead to rupture of the uterus.

At the same time, the fact that they are of common occurrence must be borne in mind, and the additional fact that many women pass safely through pregnancy and labour, even when quite obvious tumours are present. But complications may occur and become serious, or even fatal with great rapidity, and all pregnant women who are known to have palpable fibroids should be put under constant and close observation.

In all cases of the co-existence of tumours of the appendages or the uterus and pregnancy, it should be remembered also that acute symptoms if they arise are very apt to lead to premature expulsion of the contents of the uterus; so that this event occurring as the result of operation, is in the majority of instances only an anticipation of an event which is almost a certainty.

As regards the technique of the operation, the chief point to avoid is the handling and disturbance of the uterus; in the case of ovarian tumours this can be reduced to a ninimum, but with fibroids it is unavoidable. The closure of the wound should be such as will give a firm scar which will not yield under the pressure of the enlarging uterus, and to secure this the wound should always be sewn up in layers, and the sheath of the rectus well overlapped in the whole length of the incision.

### Cases of Ovariotomy during Pregnancy.

**Case 1.**—A primigravida, aged 33. Pregnancy advanced to the sixth month. A cystic swelling, movable, and not particularly tender, felt rising above the umbilicus. A sulcus could be felt between the cyst and the pregnant uterus.

The abdomen was opened, and an ovarian cyst of the left side delivered after tapping. The wound was sewn up in layers. The patient made a good recovery and went to term, but the child was stillborn.

**Case 2.**—A multipara, aged 33. Considerable difficulty was found in establishing the diagnosis of ovarian tumour, since the whole abdomen was distended by a fluctuating swelling, apparently uniform, reaching to the ensiform, one side of the swelling being contractile and the other not so. A provisional diagnosis of ovarian tumour, with a pregnancy of about six months, was made, and confirmed by tapping the non-contractile portion of the swelling with a hypodermic needle, and drawing off a small quantity of fluid, the chemical and microscopical constituents of which proved it to be ovarian in origin. A large, thin-walled cyst was removed by laparotomy, but the patient miscarried four days after the operation. Recovery was otherwise uneventful.

### Cases of Myomectomy during Pregnancy.

**Case 1.**—Primigravida, aged 40. Suffering from an abdominal tumour, complicating pregnancy. 14/6/05.

The patient stated she had missed three periods, but had noticed an enlargement of the abdomen, and been conscious of the presence of a tumour for between six and seven months.

She sought advice on account of pain and dyspnœa, caused by the presence of the tumour.

On abdominal examination the whole abdomen was found to be occupied by a hard, rounded, nodulated swelling, extending into both flanks, and distending the upper part of the abdomen. There was much difficulty in respiration due to mechanical interference with the diaphragm, due to the presence of the tumour, and a considerable amount of pain from pressure. On vaginal examination the cervix was found to be soft, and the uterus could be defined as a soft rounded swelling in the pelvis. As the difficulty in respiration and the pain were rapidly increasing, laparotomy was decided upon.

On opening the abdomen a large fibroid presented, attached by a thick pedicle to the fundus of the uterus. The pedicle was ligatured and cut through by a V-shaped incision, and the fibroid removed. The peritoneum was was then sewn together over the stump and the ligatures, and the abdomen closed.

The patient made a good recovery, and was discharged practically well three weeks later.

**Case 2.**—A primigravida, aged 34, was sent to me on account of an abdominal tumour which had suddenly become extremely painful. She had missed four periods, and sought advice on account of the increasing pain and size of the tumour.

On examination a tumour, apparently double, was felt rising to just above the level of the umbilicus. The left lobe was soft and elastic, while the right was hard and apparently solid.

Both tumours were approximately of the same shape and size, the size of the tumour corresponding to that of a four and a half months' pregnant uterus.

On opening the abdomen a tumour was found growing from the pregnant uterus, the shape of the uterus and tumour combined being that of a heart on a playing card. The uterus was rotated on its long axis, so that the right cornu which formed the left lobe of the double tumour lay in the left iliac fossa, and contained a fibroid larger than a coco-nut. The tumour in the right iliac fossa was found to be the pregnant uterus, from the right cornu of which the fibroid was growing.

The capsule of the tumour was incised, and the fibroid enucleated. The cavity left was closed with three tiers of continuous catgut sutures, and the peritoneum united over the sutured incision in the capsule.

The patient made a good recovery, and left the hospital a month after operation. Six weeks later, however, at the end of the six month, she miscarried. She made a good recovery, and has since been delivered of a full-time child without difficulty.

**Case 3.**—Primigravida, aged 29. Sought medical advice on account of pain in the pelvis, frequency and difficulty in micturition, and other pressure symptoms.

On examination the fundus of the pregnant uterus could be felt just above the brim of the pelvis. On vaginal examination the size of the uterus was found to be that of a three months' pregnancy, while growing from its posterior aspect, at about the level of the junction of the supra-vaginal portion with the body of the uterus, was felt a rounded tumour which depressed the posterior vaginal fornix, and occupied practically the whole of the space in the posterior half of the pelvis.

Owing to the rapid increase in the pain and pressure symptoms while she was under observation, immediate operation was decided on after the patient had been offered the alternative of going to term, and being delivered, if necessary, by Cæsarean section. The posterior vaginal vault was incised, and the pouch of Douglas opened. The capsule of the tumour was seized with vulsellum forceps, incised, and the fibroid gradually enucleated from its connection with the uterus, and brought down into the vagina. The raw surface left was closed by a double tier of continuous catgut sutures, and the peritoneum over-sewn. The patient made a good recovery, and I delivered her at term, after an absolutely normal labour, of a healthy female child.

She has since been delivered of a second child after a normal pregnancy and labour.

**Case 4.**—Primigravida, aged 28. This patient was between four and five months pregnant. The fœtal heart could be heard, and on the anterior surface of the fundus uteri there was felt a projecting rounded mass, obviously a fibroid, which had been increasing in size with great rapidity during the previous three weeks, and was giving rise to a considerable amount of pain.

The abdomen was opened, the fibroid enucleated, and the raw surface left sewn up, and the peritoneum over-sewn as in the previous cases. The patient made a good recovery, and was confined early in January, 1912.

In these cases, in Case 1 only was the tumour pedunculated, and in Cases 2, 3, and 4 the tumour was sub-peritoneal but sessile.

In each case symptoms were present calling for immediate relief: in Case 1 mechanical upward pressure, in Cases 3 and 4 increasing pain, and in Case 2 pain and pelvic pressure symptoms.

